# The use of technology to improve medication adherence in heart failure patients: a systematic review of randomised controlled trials

**DOI:** 10.1186/s40545-023-00582-9

**Published:** 2023-06-29

**Authors:** Chloe Cheng, Gemma Donovan, Naseer Al-Jawad, Zahraa Jalal

**Affiliations:** 1 School of Pharmacy, Institute of Clinical Sciences, University of Birmingham, Edgbaston, Birmingham, B15 2TT UK; 2 Generated Health Ltd, Mercury House, 117 Waterloo Road, London, SE1 8UL England; 3 School of Computing, The University of Buckingham, Hunter Street, Buckingham, MK18 1EG UK

**Keywords:** Medication adherence, Heart Failure, Technology, Self-care management

## Abstract

**Background:**

Heart failure is an ever-growing contributor to morbidity and mortality in the ageing population. Medication adherence rates among the HF population vary widely in the literature, with a reported range of 10–98%. Technologies have been developed to improve adherence to therapies and other clinical outcomes.

**Aims:**

This systematic review aims to investigate the effect of different technologies on medication adherence in patients with heart failure. It also aims to determine their impact on other clinical outcomes and examine the potential of these technologies in clinical practice.

**Methods:**

This systematic review was conducted using the following databases: PubMed Central UK, Embase, MEDLINE, CINAHL Plus, PsycINFO and Cochrane Library until October 2022. Studies were included if they were randomised controlled trials that used technology to improve medication adherence as an outcome in heart failure patients. The Cochrane Collaboration's Risk of Bias tool was used to assess individual studies. This review was registered with PROSPERO (ID: CRD42022371865).

**Results:**

A total of nine studies met the inclusion criteria. Two studies showed statistically significant improvement in medication adherence following their respective interventions. Eight studies had at least one statistically significant result in the other clinical outcomes it measured, including self-care, quality of life and hospitalisations. All studies that evaluated self-care management showed statistically significant improvement. Improvements in other outcomes, such as quality of life and hospitalisations, were inconsistent.

**Conclusion:**

It is observable that there is limited evidence for using technology to improve medication adherence in heart failure patients. Further studies with larger study populations and validated self-reporting methods for medication adherence are required.

**Supplementary Information:**

The online version contains supplementary material available at 10.1186/s40545-023-00582-9.

## Keypoints

- The evidence in this review shows the potential for technology use in practice to improve health outcomes for patients with heart failure. Still, there is uncertainty in the evidence on its impact on medication adherence.
- Technologies presented in the studies are generally acceptable for participants, which is informative for future implementations into practice.
- There was a lack of high-quality studies investigating technology's use on medication adherence in heart failure. More extensive studies with improvements to study design are needed to ultimately conclude the effects on medication adherence.

**Supplementary Information:**

The online version contains supplementary material available at 10.1186/s40545-023-00582-9.

## Introduction

Heart failure (HF) is a debilitating chronic condition with high morbidity and mortality affecting over 900,000 people in the UK, with a higher prevalence above age 65 [1–3]. Similarly, the population of HF patients per year continues to increase [4]. HF patients are roughly equally split between those with reduced Ejection Fraction (HFrEF) and those with preserved or mildly reduced Ejection Fraction (HFpEF or HFmrEF) [2]. All three types usually require pharmacological therapy to manage symptoms, co-morbidities, and heart function [4]. These include diuretics, Angiotensin-Converting Enzyme (ACE) inhibitors, beta-blockers, and Angiotensin Receptor Blockers (ARBs) [4]. Medication Adherence (MA) is defined as "the extent to which a patient’s action matches the agreed recommendations" [5]. MA rates among the HF population vary widely in the literature, with a reported range of 10–98% [6, 7]. Low adherence rates are associated with worse cardiac event-free survival, all-cause mortality, cardiovascular hospitalisations, and a more significant symptom burden [8, 9]. Studies have shown a greater rate of non-adherence in the elderly population, potentially due to co-morbidities and polypharmacy [10]. On the other hand, high MA is linked to fewer HF symptoms, lower rates of hospitalisation, and fewer deaths [9].

MA is affected by multiple factors, including patient beliefs, socioeconomic influences, and the type of prescribed therapies [11]. Educational interventions to address these issues have been shown to be effective [11]. However, this does not guarantee that medications will be taken, especially if regimens require multiple doses each day or a patient has various medications to take. Interventions to tackle the lack of medication adherence have been the focus of technological advancements, for example, mobile applications, electronic pill boxes, automated telephone calls, and messaging [11]. Previous trials using these technologies have shown significant increases in refilling medications, specifically in patients with cardiovascular diseases (CVD) such as hypercholesterolaemia, hypertension, and coronary heart disease [11].

Systematic reviews have previously evaluated specific technological interventions such as mobile health and application (app) technology; however, they only evaluated CVD as a whole [12]. Even in systematic reviews that studied technology use in HF, MA was not the primary objective [13]. There is also limited evidence assessing MA specifically in HF [13]. Hence, this systematic review aims to assess the impact of technology on MA in HF patients.

## Aim

1. To investigate the effect of different technologies on medication adherence in patients with heart failure.
2. To determine the impact of different technologies on other clinical outcomes for heart failure patients and examine the potential of these technologies in clinical practice.

## Methods

This systematic review was conducted according to PRISMA guidelines and was registered with PROSPERO (registration ID: CRD42022371865).

### Search strategy

An electronic search of the following databases was conducted for research articles: PubMed Central UK, Embase (Ovid), MEDLINE (Ovid), CINAHL Plus (EBSCO), PsycINFO (Ovid) and Cochrane Library, from 2000 until October 2022. The keywords' heart failure', 'medication adherence', and 'technology' were used in this review. Filters for Randomised Controlled Trials (RCTs) and the English language were applied. The MeSH term's function was utilised to generate other keywords for each database (see example search strategy in Additional file 1: Table S1. Reference lists of included studies were reviewed to identify additional relevant RCTs.

### Study inclusion criteria

Studies that were RCTs and included technology as an intervention to improve adherence to HF medication were included. Studies were required to have a usual care control group. Studies with patients aged 18 or over were included, and participants could be of any class of the New York Heart Association (NYHA) classification of HF. Studies needed to include patients taking at least one medication related to HF and have a precise MA outcome measure. Studies involving patients under the age of 18 and non-RCTs: observational studies, systematic reviews, meta-analyses, grey literature, abstract available only, unpublished studies, and quasi-randomised trials, were excluded, along with those that were not in the English language.

### Study selection and data extraction

All the identified studies were imported and intensively analysed to remove duplicate records manually. The first author (CC) screened all titles and abstracts using the inclusion criteria. This was checked by another author (ZJ). All researchers screened the full texts of the remaining articles independently to identify eligible studies (CC, GD, and ZJ). Any studies that displayed uncertainty were discussed among all the authors to reach a consensus. All relevant studies underwent data extraction for country of origin, number of study arms, intervention description, length of intervention, outcome measures, sample size, and results for comparison.

### Outcomes assessed

The primary outcome assessed was MA in HF. Secondary outcomes were quality of life, self-care/self-management behaviours, HF knowledge, number of hospital admissions, mortality, health status, self-efficacy, depression, clinical follow-up attendance, patient satisfaction, physiological measures (e.g., NT-proBNP and HbA1C), and physical activity.

### Quality assessment

Quality assessment was conducted using the Cochrane Collaboration's Risk of Bias Tool to assess each study's risk of bias according to the following seven domains: random sequence generation, allocation concealment, blinding of participants and personnel, blinding of outcome assessment, incomplete outcome data, selective reporting bias, and other bias [14]. Each study's domains were allocated to low-risk, unclear-risk, or high-risk of bias [14]. The risk of bias summary graphs were generated using RevMan software version 5.4.

## Results

### Search results

**Fig. 1:**
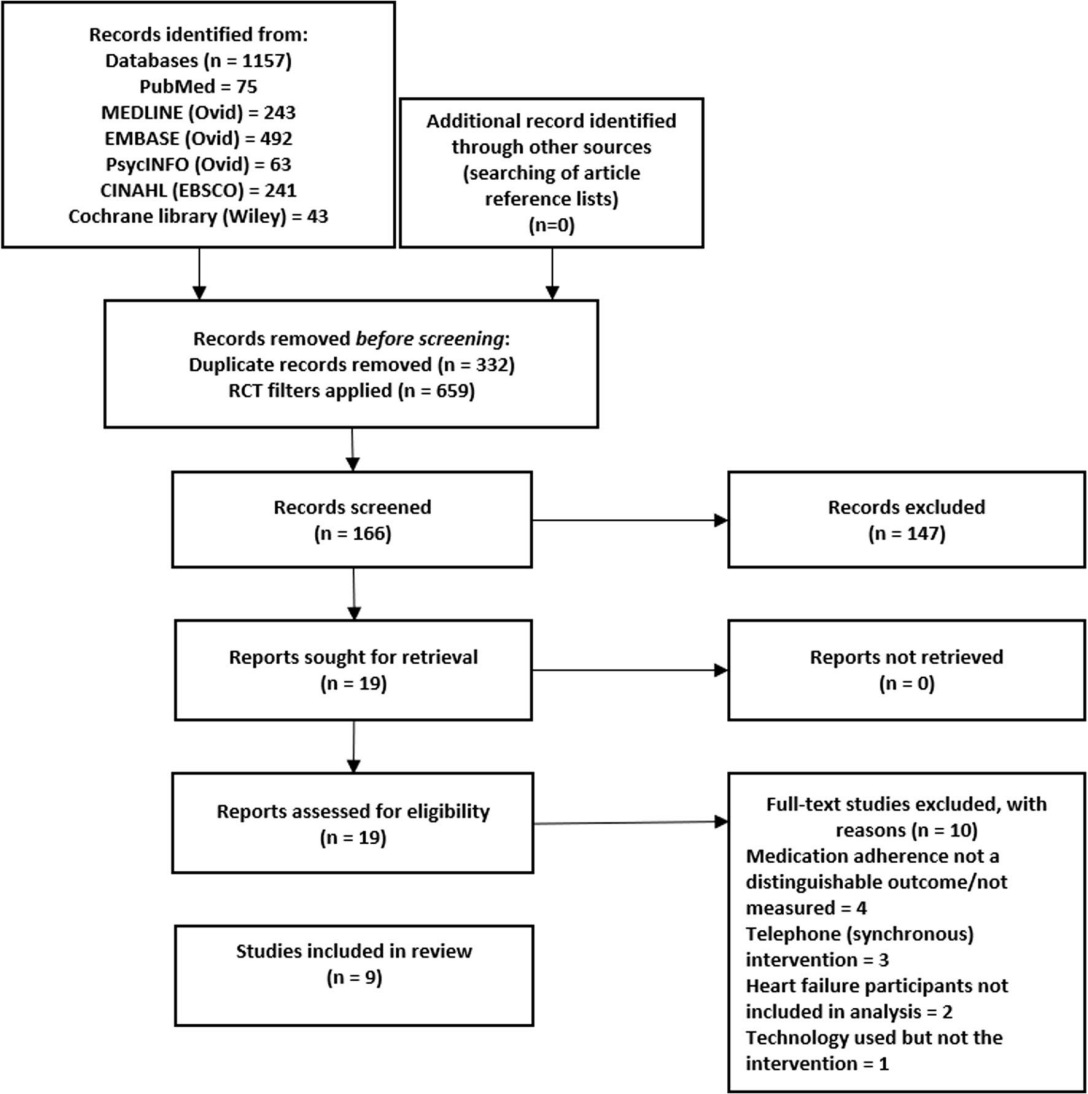
Preferred Reporting Items for Systematic Reviews and Meta-Analyses (PRISMA) flow chart of the study selection process

The initial search of databases identified 1157 records. No additional studies were identified from the reference lists of included studies. Once duplicates were removed, and RCT filters were applied, 166 studies were screened using titles and abstracts. After exclusion, 19 studies were screened using full-text, and ten studies were excluded due to reasons listed in the PRISMA diagram in Fig. 1. Subsequently, nine studies were included [15–23]. Six of the nine studies were RCTs [18–23], two studies were pilot RCTs [15, 16], and one study was a randomised controlled feasibility trial [17]. A meta-analysis was considered; however, because of the heterogeneity of the outcome measure tools (specifically the self-reporting tools used to measure medication adherence), and variations in other factors such as sample size and intervention length, this made it difficult to bring together an appropriate statistical analysis.

### Study characteristics and design

Included studies were published between 2004 and 2022. Seven studies were conducted in the United States [15–17, 19, 20, 22, 23]. One study was conducted in the Netherlands and another in Argentina [18, 21]. All studies involved one or more intervention arms against a control arm [15–23]. All study controls were usual care, defined as participants undergoing follow-up without the intervention [15–23]. However, three studies gave their usual care participants the intervention technology dice to either measure medication adherence as a study outcome or programmed it to be silent [16, 17, 19] All nine studies used a similar study design [15–23]. The sample size varied between 29 and 382 participants, with 1032 participants and intervention duration ranging from 28 days to 12 months [15–23]. Table 1 provides a summary of study characteristics.

**Table 1:** Study characteristics for the included studies

Study	Study design	Sample size	Intervention	Control group	Intervention arms	Length of intervention	Primary outcomes	Secondary outcomes
Hale et al. [15], USA	Randomized controlled pilot study	Intervention = 13 Control = 16 (n = 29)	MedSentry remote medication monitoring system- medication contained in individual bins with participants alerted when it is time to take it	Usual care: using their usual medication reminder method	2 arms (control versus intervention)	90 days	• Medication adherence • Health status • Quality of life • Emergency department (ED) visits and hospitalizations	Not applicable
Gallagher et al. [16], USA	Randomized controlled pilot study	Intervention = 20 Control = 20 (n = 40)	Telemonitoring (in combination with the GlowCap system- patients told they may be contacted for monitoring in intervention group	Usual care with passive monitoring	2 arms (control versus intervention)	30 days	• Adherence to loop diuretics	• 30-day all cause readmission • Attendance at follow-up • Acceptability of telemonitoring
Goldstein et al. [17], USA	Randomized controlled feasibility trial	Smartphone (n = 30) Intervention = 15 Control = 15 Container (n = 30) Intervention = 15 Control = 15 (n = 60)	Telehealth intervention (electronic pill box) and m-health intervention (app on smartphone)	Usual care with intervention device that did not provide reminders—"silent condition"	2 × 2 arms (two control versus intervention) 1.Pillbox silent 2.Pillbox reminding 3.Smartphone silent 4.Smartphone reminding	28 days	• Medication adherence • Mastery of intervention (participants ability to use their given device and its functions) • Device ratings	Not applicable
Boyne et al. [18], Netherlands	Randomised controlled trial	Intervention = 197 Control = 185 (n = 382)	Telemonitoring device (HealthBuddy)- connected to a landline. Participants answered daily questions about symptoms, knowledge, and behaviour by touching one of the device keys	Usual care with easy access to a HF nurse and 4 outpatient clinic visits during follow-up	2 arms (control versus intervention)	12 months	• Disease-specific knowledge • Disease-specific self-care • Disease-specific self-efficacy • Disease-specific adherence to therapy	Not applicable
Wu et al. [19], USA	Randomised controlled trial	TPB + MEMS group = 27 TPB = 27 Control = 28 (n = 82)	Theory of Planned Behaviour (TPB) AND Medication Event Monitoring System (MEMS) feedback	Usual care	3 arms 1.TPB and MEMS (PLUS) 2.TPB (LITE) 3. Usual care (Control)	9 months	• Cardiac event-free survival • Quality of life • Medication adherence	Not applicable
Felker et al. [20], USA	Randomised Controlled Trial	Intervention = 92 Control = 95 (n = 187)	Mobile Health (mHealth) intervention- educational skill-based teaching for medication management	Usual care	2 arms (intervention versus control)	3 months (6 months follow-up as well)	• Change in mean daily step count from baseline through 3 months	• Change in medication adherence • Quality of life • Physiological measure of disease status (NT-proNBP, HbA1c) • Metabolic profiling
Yanicelli et al. [21], Argentina	Randomised Controlled Trial	Intervention = 20 Control = 20 (n = 40)	Home telemonitoring system (HTS)- application (app) that collects physiological measures and symptoms, with an educational and messaging function	Usual care	2 arms (intervention versus control)	90 days	• Changes in self-care • Changes in treatment adherence	• Rehospitalisation
Young et al. [22], USA	Randomised Controlled Trial	Intervention = 54 Control = 51 (n = 105)	Electronic pill organizer reminder	Usual care with standard discharge teaching and scheduled follow-up doctor's appointment	2 arms (intervention versus control)	12-week SM training 3 + 6-month follow-up	SM adherence: • Frequencies of daily weighing • Following low-sodium diet • Taking prescribed medication • Exercising • Attending follow-up appointments • Clinical biomarkers • All-cause readmissions • ED visits	• Physical activity
Ross et al. [23], USA	Randomised controlled trial	Intervention group = 54 Control group = 53 (n = 107)	SPPARO (System Providing Access to Records Online): • Web-based electronic medical record • Web-based educational guide • Web-based messaging system	Usual care	2 arms (control versus intervention)	12 months	• Self-efficacy	• Patient satisfaction • Adherence (medication and general)

### Technology characteristics

A range of technologies was used in the included studies: Remote medication monitoring systems, telemonitoring devices, electronic pill boxes, apps/text-messaging, electronic pill organiser reminders, and web-based technologies [15–23]. Six studies used their technology interventions as a form of patient education to increase MA [16, 18–21, 23]. The other three studies used medication reminders via the technologies to improve MA [15, 17, 22].

Three studies used single interventions (their respective technology devices). Still, participants were contacted by telephone if they were identified as non-adherent to their medication [15, 16] or classed as high-risk based on their adherence data or answers about symptoms, HF knowledge, and medication-taking behaviours [18]. One study used medication event monitoring system (MEMS) feedback in addition to a counselling intervention known as the 'Theory of Planned Behaviour (TPB)' [19]. Another study used a home telemonitoring system entailing an app with multiple functionalities such as physiological measure monitoring and education [21]. One study used various interventions such as face-to-face self-management sessions, telephone reinforcement sessions, and a self-management toolkit (with devices such as a weight scale and an electronic pill organiser reminder) [22]. Web-based technology in one study created a Web-interface for participants with three components: patient's medical record, education guide, and messaging system with practice nurses [23]. Goldstein et al. used a 2 × 2 study design, where a telehealth intervention versus a control group took place alongside a mobile health intervention against a control group [17]. The mobile health intervention in Felker et al. involved two aspects: personalised text messages to summarise physical activity performance and goal setting and an electronic tool to provide educational materials on medications [20].

### Assessment of medication adherence

MA was measured in various ways across the studies. Six studies solely used self-reporting tools or questionnaires [15, 18, 20–23]. Two studies used the modified Morisky scale or questions derived from it [21, 23]. Other self-reporting measures included an adherence questionnaire from Voils et al. [20], the 'Heart Failure Compliance Scale' [18], a self-reporting question from the Medical Outcomes Study (MOS) [15] and a self-reporting question of the day's medication was missed in the last seven days [22]. Two studies exclusively used a dose count from the technological intervention device as their measure outcome [16, 19]. One study used two outcome measures depending on the intervention- the telehealth groups used a dose count percentage, whilst the mobile health groups used a self-reported measure [17]. The study that used a question from MOS also used a dose count from the intervention device, but only for the intervention group [15]. The outcome measures for each study are shown in Table 2.

**Table 2:** The effectiveness of technology on medication adherence

Study and Country	Method of measuring medication adherence	Effect on medication adherence	P-value
Hale et al. [15], USA	One question from Medical Outcomes Study (MOS) MedSentry data- counted as 'missed if not taken within 1 h	At 90 days follow-up, Intervention group medication adherence was 69% (n = 9) versus 73% (n = 13) measured using a MOS question Medication adherence in the intervention group did not improve from baseline of 98.7% versus 94.2% at 90 days follow-up using data from the MedSentry device	0.610
Gallagher et al. [16], USA	Percentage of days where number of correct doses were taken (Mann–Whitney U test used to compare adherence percentages between groups)	Adherence to diuretics (> 88% doses taken) was 29.4% (n = 5 participants) in the intervention group at 30 days follow-up, compared to 36.8% (n = 7) in the control group	0.640
Goldstein et al. [17], USA	Pillbox opening recording Smartphone- electronic self-report (patients recorded their medication-taking on a log available on the medication adherence app – options for each event were taking a medication or skipping it)	At 28 days follow-up, intention-to-treat analyses showed patients in both intervention groups adhered to their medications 79% of the time, a mean of 84% for pillbox and 73% for smartphone intervention. This is compared to a mean of 78% adherence in the passive medication device groups (from 76% in pillbox and 79% in smartphone) Per protocol analysis found that the intervention groups had a mean adherence rate of 85% versus a mean of 80% in the control groups	0.480
Boyne et al. [18], Netherlands	Heart Failure Compliance Scale (Higher scores indicate better adherence)	Perceived importance of medication increased after 6 (93.5% intervention versus 88% control, p = 0.012) and 12 months (93.5% intervention versus 89.8% control, p = 0.037) Estimated medication adherence based on X was greater in the intervention at 12 months (100% intervention versus 98.7% control) but was not found to be statistically significant)	0.107
Wu et al. [19], USA	MEMS (> 88% recorded opening as scheduled = adherent; < 88% = non = adherent)	PLUS patients had significantly better medication adherence versus control group at 2 months follow-up (82% intervention versus 59% control, p = 0.05) and at 9 months follow-up (74% intervention versus 36% control, p = 0.012)	0.021
Felker et al. [20], USA	Adherence questionnaire developed by Voils et al	Medication adherence did not change from baseline to 3 months (Least Squares (LS)-mean change: –0.08 in mHealth vs –0.15 in usual care; LS-mean difference = 0.07; 95% CI: –0.12, 0.26)	0.470
Yanicelli et al. [21], Argentina	Morisky Modified Scale (MMS) questionnaire (Higher scores indicated better adherence)	Mean MMS overall scores in 3 months follow-up were 4.73 (intervention) and 4.73 (control)	0.800
Young et al. [22], USA	Self-reported number of days where medication had been missed in the previous 7 days	Intervention group also reported significantly fewer days missing any doses of prescribed medication Mean number of days for any missed medication in the previous 7 days was 0.39 in the intervention group versus 0.81 (control) at 3 months and 0.26 (intervention) versus 0.80 (control) at 6 months. Estimated marginal mean was 0.30 (intervention) versus 0.80 (control). 95% CI: -0.51 (-0.97, -0.05)	0.030
Ross et al. [23], USA	Questions derived from Morisky Scale	At 6 months, medication adherence scores were 3.5 (Intervention) versus 3.4 (control), with a CI of + 0.1 (-0.2, 0.4). At 12 months, scores were 3.6 (intervention) versus 3.4 (control) with a CI of + 0.2 (-0.1, 0.6)	0.150

### Effect of medication adherence

Eight of the nine studies showed improvement in MA following the intervention [15–19, 21–23] (Table 2). The remaining study is unclear as they did not report the direction of their scoring system for medication adherence [20]. However, only two studies showed a statistically significant improvement in MA. The study by Wu et al. used MEMS feedback as an educational tool for the patient. It showed 74% of the intervention group adherent to medication versus 36% in the control group [19]. The study by Young et al. used an electronic pill organiser reminder as a medication reminder intervention and found improvement with a marginal mean of 0.8 days if any medication was missed in the last seven days for the control group versus 0.3 days in the intervention group [22].

### Effect on clinical and non-clinical outcomes

A table of other clinical outcomes examined in included studies can be found in Additional file 1: Table S2. These included: quality of life, self-care/self-management behaviours, HF knowledge, number of hospital admissions, mortality, health status, self-efficacy, depression, clinical follow-up attendance, patient satisfaction, physiological measures, and physical activity.

#### Quality of life

Table 3 shows only one of the three studies that assessed the quality of life showed significant improvement in quality of life [20]. Another study showed significance, but the quality of life was worse in the intervention group than in control [15]. A-value was not available in the previous research [19].

**Table 3:** Quality of life outcome measures and results

Study	Measurement of quality of life	Results	P-value(s)
Hale et al. [15], USA	Minnesota Living with Heart Failure Questionnaire (Higher score indicates worser quality of life)	Mean score for quality of life at 90 days follow-up for intervention group (PLUS) was 62.2 for intervention group versus 28.2 in control group	0.002
Wu et al. [19], USA	Minnesota Living with Heart Failure Questionnaire (Higher score indicates worser quality of life)	Mean score for quality of life at 90 days follow-up for intervention group (PLUS) was 32.9 for intervention group versus 40.1 in control group	0.464
Felker et al. [20], USA	Kansas City Cardiomyopathy Questionnaire	Significant difference in quality of life (difference 1.1 with 95% CI [1.4, 9.6]). Higher in intervention	0.009

#### Hospitalisation

Hospitalisations showed variations in the results, with two of five studies showing a significant difference in the number of hospital admissions [15, 19] (Table 4).

**Table 4:** Hospitalisation outcome measures and results

Study	Clinical outcomes	Results	P-value(s)
Hale et al. [15], USA	Hospitalisations ED visits	Number of participants who had one or more HF-related hospitalisations were 4 in intervention group versus 1 in control group and non-HF related hospitalisations were 1 in intervention group versus 4 in control group after 90 days. All-cause hospital admissions were significant, with 1 in intervention group versus 4 in control group Number of participants who had one or more HF-related ED visits were 1 in intervention group versus 3 in control group and non-HF related ED visits were 3 in intervention group versus 4 in control group after 90 days. All-cause ED visits were not significant, with 3 in the intervention group versus 6 in the control group	0.340 (HF), 0.340 (non-HF), **0.040** (all-cause) 0.600 (HF), 0.990 (non-HF), 0.680 (all-cause)
Gallagher et al. [16], USA	30-day all cause readmission	Number of readmissions were 6 in the intervention group and 4 in the control group after 30 days follow-up	**0.720**
Wu et al. [19], USA	Cardiac event-free survival (Including ED visit, hospitalization, death)	Event-free survival was significantly longer for the patients in both intervention groups than the control	**0.010**
Yanicelli et al. [21], Argentina	Rehospitalisation (from electronic medical record)	Number of rehospitalisation were 0 for intervention group and 2 in control group after 3 months follow-up	**0.500**
Young et al. [22], USA	All-cause readmissions ED visits	Number of participants readmitted to hospital was 10 for intervention group versus 3 in the control group Number of participants admitted for ED visits at 90 days were 9 for intervention group versus 11 in control group	**0.088**

Bolded p-values represent the significance values used as part of the synthesis. For fairness of comparison, all-cause hospitalisation was used in Hale et al, to encompass all hospitalisations regardless of their relation to heart failure

#### Health-status

One study evaluated health status and did not show significance [15].

#### Self-care management

Four studies showed significant improvement in self-care/self-management or general adherence to self-care [18, 21–23] (Table 5).

**Table 5:** Self-care/general adherence outcome measures and results

Study	Clinical outcome and measurement	Results	P-value(s)
Boyne et al. [18], Netherlands	European Heart Failure Self-Care Behaviour Scale (EHFScB) (Lower scores indicate better results)	Mean self-care score at 12 months follow-up was 17.4 for intervention group versus 20.8 in control group	< 0.001 (uncorrected) ** < 0.001 at T = 12 (corrected)**
Yanicelli et al. [21], Argentina	European Heart Failure Self-Care Behaviour Scale (EHFScB) (Higher scores indicate better results)	Mean self-care score at 3 months follow-up was 80.03 for intervention group versus 69.43 in control group	**0.004**
Young et al. [22], USA	Other self-management adherence	Mean number of days for weighing self per week was 4.8 in the intervention group versus 1.9 (control) at 3 months and 4.6 (intervention) versus 1.5 (control) at 6 months. Estimated marginal mean was 4.7 (intervention) versus 1.7 (control). 95% CI: 2.98 (2.10, 3.86) Mean number of days for following low-sodium diet per week was 5.6 in the intervention group versus 3.1 (control) at 3 months and 5.1 (intervention) versus 2.3 (control) at 6 months. Estimated marginal mean was 5.3 (intervention) versus 2.7 (control). 95% CI: 2.62 (1.74, 3.50) Mean number of days for exercising per week was 5.4 in the intervention group versus 3.4 (control) at 3 months and 4.5 (intervention) versus 3.1 (control) at 6 months. Estimated marginal mean was 4.9 (intervention) versus 3.3 (control). 95% CI: 1.66 [0.79, 2.53]	** < 0.005** ** < 0.005** ** < 0.005**
Ross et al. [23], USA	General Adherence Scale from the Medical Outcomes Study (MOS) (Higher scores indicate better adherence)	Mean score for general adherence was 85 in the intervention group versus 78 in control group. 95% CI + 6.4 (1.8, 10.9)	**0.020**

Bolded P-values represent the significance values used as part of the synthesis. For fairness of comparison, corrected values (where applicable to the study) were used as opposed to uncorrected values

#### Patient satisfaction with the intervention

Two studies evaluated device rating/patient satisfaction as an outcome towards the intervention- one study observed that patients statistically significantly preferred m-health over telehealth [17]. The other resulted in 88% of participants rating the device as somewhat or very easy to use [16]. Ross et al. measured patient satisfaction with doctor patient-communication, with improvements seen in some domains, but which were not significant [23].

#### Other outcomes

One study assessed depression and did not show improvement [15]. Follow-up attendance did not improve in another study [16]. Boyne et al. showed statistically significant improvement in disease-specific knowledge at the end of follow-up [18]. Three studies assessed self-efficacy, two of which did not improve [18, 23], while one did [22]. Two trials measured physical activity- one showed significant improvement in mean daily step count [20], whilst the other did not in activity minutes, calories burnt, or daily activity counts [22].

Lastly, two studies evaluated physiological parameters such as HbA1c and NT-proBNP with no significant differences shown [20, 22]. One of those studies also evaluated metabolic profiling with significant changes observed for the intervention in 13 metabolites [20].

### Risk of bias of included trials

**Fig. 2:**
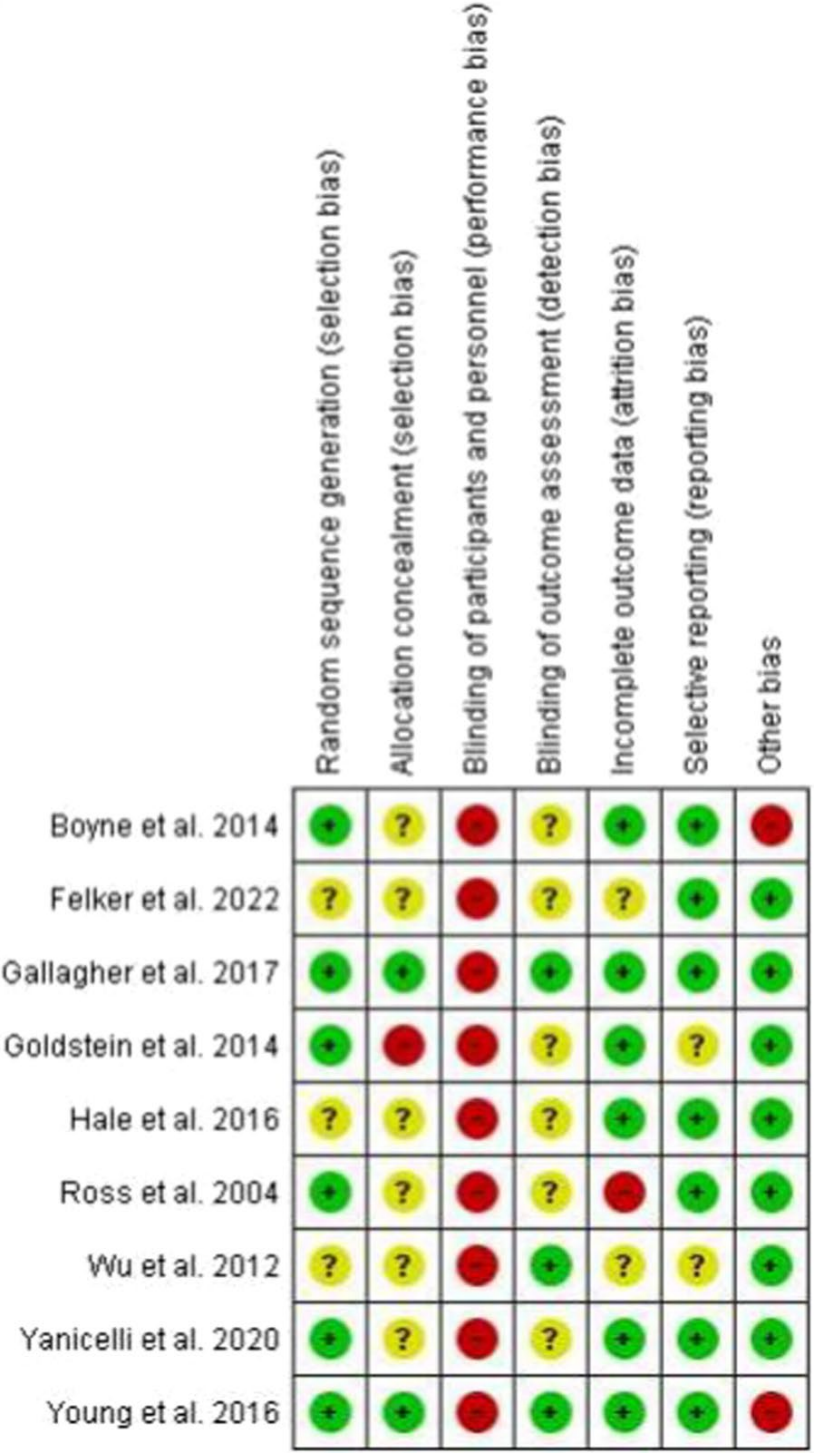
Risk of bias summary graph and the outcome of each domain for included studies

**Fig. 3:**
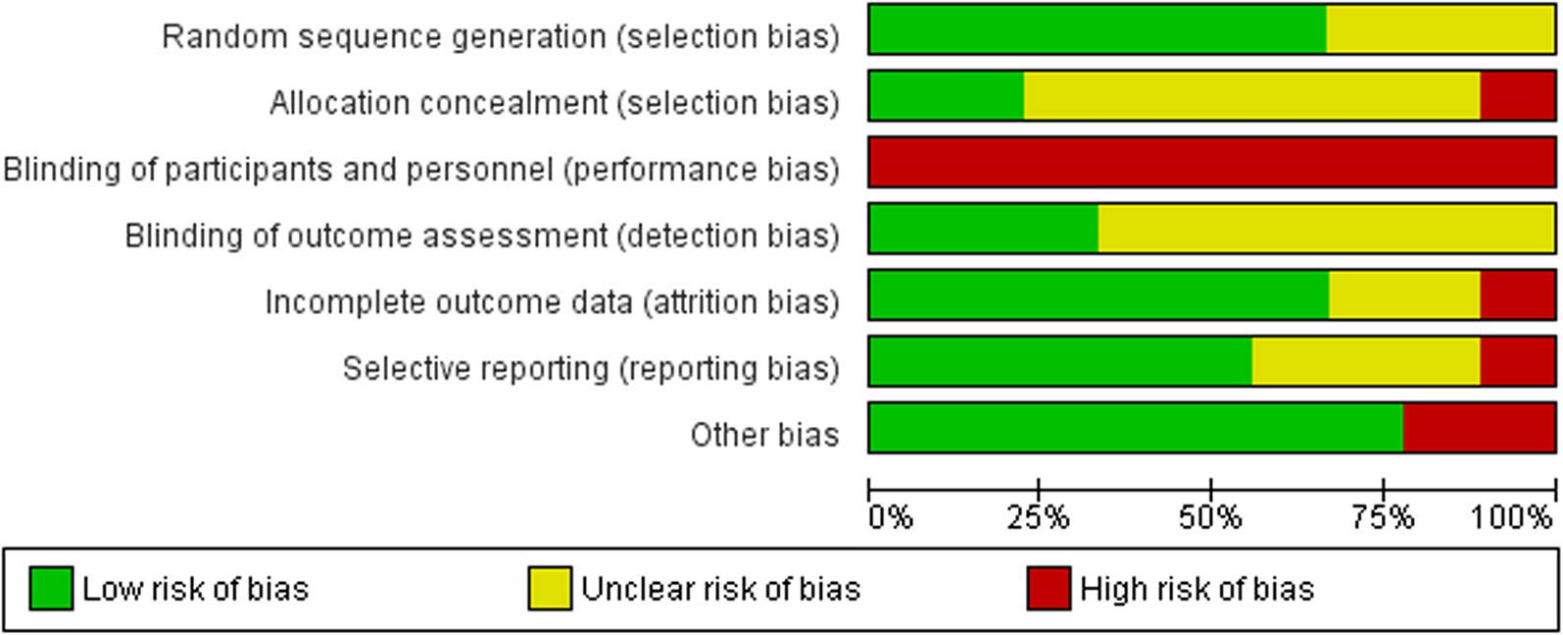
Risk of bias graph of included studies with percentages for each risk of bias domain

The risk of bias summary graphs were generated using RevMan 5.4 software (Figs. 2, 3). Six studies reported appropriate random sequence generation [16–18, 21–23], with the other three showing unclear risk [15, 19, 20]. In contrast, six studies showed unclear allocation concealment [15, 18–21, 23], two with low risk [16, 22], and one reporting a high risk of bias [17]. Blinding of participants and personnel showed a high risk of bias for all studies as it wouldn't have been possible to blind participants, due to the type of intervention involved. However, blinding personnel could have been possible, with only one of the studies mentioning that the study team members were blinded to group assignments [16]. Six studies reported an unclear risk of bias for blinding of outcome assessment [15, 17, 18, 20, 21, 23], with the other three showing low risk [16, 19, 22]. Assessment of incomplete outcome data showed six studies with a low risk [15–18, 21, 22], two with unclear risk [19, 20], and one study being a high risk for attrition bias [23]. Selective reporting assessment displayed seven studies with a low risk of bias [15, 16, 18, 20–23] and two studies for unclear risk [17, 19]. Lastly, seven studies exhibited a low risk of other bias [15–17, 19–21, 23], specifically on whether the tools they used as outcome measurements for MA were reported as being validated, known to be validated, or showed a quantitative measure of adherence. The other two studies did not show this, therefore, were classed as high risk for bias [18, 22].

## Discussion

This systematic review aimed to determine technology's impact on MA in HF patients. This review involved nine RCTs with a total of 1032 participants and used some form of technology to support MA and evaluate this as an outcome [15–23]. Two RCTs showed statistically significant improvement in MA, both of which used mixed interventions [19, 22]. Six RCTs showed improvement, but results were not statistically significant [15–18, 21, 23], and one RCT showed unclear improvement [20]. Depending on the study, the technologies provided different purposes- patient education or medication reminders (with no known comparisons on which is more effective in MA). To our knowledge, no other reviews assess technology for MA, specifically in HF.

It is worth noting that the two RCTs that showed significant improvement did not use technology in isolation [19, 22]. They were used in conjunction with non-technology components, for example, synchronous communication with healthcare professionals (HCPs), remote logging of symptoms, and behavioural change therapy (TPB) [19, 22]. Multi-disciplinary involvement was common in most studies [16, 18, 19, 21–23]. Previous evidence shows that combined approaches are more likely to improve MA in HF patients, specifically in older adults [24]. This supports the notion that technology if used in practice, should be part of a multi-disciplinary approach to the care of an HF patient.

Where similar improvements have been seen in past reviews for specific technologies in CVD, most have attributed a lack of significant improvement to small sample sizes [12, 25]. Our findings also showed two pilot studies [15, 16], one feasibility study [17], and four studies that had a relatively small sample size [19, 21–23]. These studies noted they were not sufficiently powered to test the intervention's actual effect. In addition, limited research assesses MA technology use in HF. This was observed in mobile health-specific reviews by Coorey et al. and Al-Arkee et al., both only included one RCT relating specifically to HF and commented on small sample sizes as a limitation [12, 25].

MA was assessed mainly using self-reporting tools or questionnaires in seven studies [15, 17, 18, 20–23]. Some tools, however, were not validated or widely known [18, 20, 22]. Whilst self-reporting methods provide a cost-effective approach to predict clinical outcomes, they have the potential to produce the 'Hawthorne effect' whereby certain behaviours evaluated in studies differ between participants due to their awareness of being assessed [26, 27]. This leads to overestimations of proper adherence [26, 27]. One study in our review exemplified this, which attributed overestimations to 'socially acceptable answers' [18], and two others note self-reporting use in various outcomes as a limitation [15, 17]. MEMS/digital monitoring- used in three studies [16, 17, 19], is considered a more accurate way of measuring MA [28]. However, it does not guarantee the correct dose is taken. Hence, results should be interpreted with caution.

The study population of some studies may have contributed to potential ceiling effects observed in this review. For instance, one study reported a baseline adherence of 98% [18], leaving little room for the intervention to produce a significant effect. Five of the studies reported using the majority of already adherent/motivated patients [16–19, 22], where it was unclear if this was intentional (except Goldstein et al. which attributed this to their recruitment methods). Therefore, this largely excluded the non-adherent population- the ideal targets of these interventions. Participant recruitment methods including advertisements and mailing, may have automatically pooled these patient cohorts [17]. Previous literature suggests that a willingness to participate in trials through such methods voluntarily can be indicative of the motivation and education the patient already has [29]. Therefore, the selection of these patients is more likely to result in ceiling effects. The opposite has been suggested for non-adherent patients, where significant differences are more likely to be seen [30].

Despite all trials assessing a range of other clinical outcomes, there was much variation in the results, especially for the quality of life and hospital admissions. These findings are consistent with a review from Allida et al., which evaluated the use of mobile health in HF patients- limited evidence was found for the effectiveness of technology interventions on quality of life and hospital admission [13]. Variations for these two outcomes (shown in Tables 3 and 4) could be explained by the heterogeneity of the measurement tools used [13]. Our self-care/management and general adherence results differed from Allida et al., where all trials that assessed it showed statistical significance, mainly using validated or well-known tools [18, 21–23]. This demonstrates these technologies' potential to impact remote non-pharmacological care in HF positively. Another review on the use of smartphones in healthcare applications raised the importance of technology in providing education, self-management, and remote monitoring [31]. This links to the multi-intervention aspects of most studies in our review that may have contributed to improvements in self-care/management and general adherence. Three out of four trials involving self-care used educational-based technologies [18, 21, 23], and two of those studies monitored patient symptoms via the technologies [21, 22]. Therefore, these technologies must have multiple functions to improve MA and non-pharmacological behaviours.

Only four RCTs assessed patient satisfaction with the intervention technology [15–17, 21]; however, all of them showed most participants finding their technologies easy to use or helpful for their treatment plans. These results are useful for informing technology utilisation in a clinical setting, specifically in a disease population with an older age demographic (all studies showed participants over the age of 50), and where previous research has suggested links between HF patients and susceptibility to cognitive impairment [32]. It is worth noting all studies did not explicitly state the degree of cognitive impairment in their patient characteristics but stated the need for their technologies to be appropriate for them.

Our findings accord with a cardiac tele-rehabilitation review which showed the technologies' high usability, utility, and acceptability, especially in the COVID-19 climate [33]. The review also noted potential preferences for types of technologies [33], but they still appeared usable and satisfactory to patients. A study in our review observed the preference for mobile health over telehealth due to the easy integration of the mobile app into their daily routines [17]. This indicates that patients may prefer technologies that are easy to use and are already familiar. A previous review on mobile health notes that portability and multi-feature access (such as viewing patient records and monitoring) is helpful for patients and their healthcare providers [34]. Additionally, tailored approaches based on the individual's needs and behaviours should be considered [35], particularly motivation- a known contributor to adherence [36], which was not addressed in most studies.

### Strengths and limitations

This systematic review was conducted in accordance with PRISMA guidelines to reduce researcher bias. However, it was often difficult to ascertain if any improvements in MA and other outcomes were solely due to the technology or the other non-technology intervention components. Other studies that may have been relevant were excluded, such as grey literature, non-RCTs, and studies not in English. However, we used RCTs, which are well-known to place at the highest level of the hierarchy of evidence [37]. Some studies tested the intervention on a certain number of medications; for e.g., Wu et al. provided MEMs feedback for only one of the medications [19]. HF patients take multiple medications; therefore, total adherence may have differed.

### Recommendations for policy and practice

Technology can potentially improve MA in HF patients; however, our review has shown insufficient evidence for this. The RCTs included in this review showed uncertain and inconsistent results for the quality of studies and effectiveness of the technologies. On the other hand, these technologies were generally easy to use or helpful for patients. Therefore, recommendations for clinical practice cannot be made without solid evidence from good-quality studies.

### Future research

Our findings from this review indicate the need for further research. Future RCTs should be sufficiently powered, primarily targeting non-adherent patients, and use blinded outcome assessors. Using validated self-reporting tools, in addition to electronic monitoring, should be used together to increase the accuracy of results. Future studies should continue to assess usability and patient satisfaction with technologies and explore the most effective mechanisms for supporting MA, such as whether providing education or medication reminders are more effective. Using similar validated self-report tools will allow future systematic reviews to include meta-analyses to generate more robust conclusions on intervention effectiveness.

## Conclusion

Evidence for the effectiveness of technology in medication adherence is currently weak. Whilst it has indicated positive improvements for some outcomes, particularly self-care, further evidence is needed for its impact on MA. More powered trials that include larger sample sizes and mostly non-adherent cohorts are required to build on existing studies and inform the future incorporation of technology into routine clinical practice.

## Supplementary Information

**Additional file 1: Table S1.** Example search strategy- EMBASE. **Table S2.** Other clinical outcomes and their effects.

## Data Availability

Further supplementary material is available on request.

## Abbreviations

HF: Heart failure
HFrEF: Heart failure with reduced ejection fraction
HFpEF or HFmrEF: Heart failure with preserved or mildly reduced Ejection Fraction
ARBs: Angiotensin Receptor Blockers
MA: Medication adherence
app: Mobile health and application
NYHA: New York Heart Association
RCTs: Randomised Control Trials
MEMS: Medication event monitoring system
HCPs: Healthcare professionals
