# Supplementary material for: The use of technology to improve medication adherence in heart failure patients: a systematic review of randomised controlled trials
Source: J Pharm Policy Pract. 2023 Jun 29;16:81. doi: 10.1186/s40545-023-00582-9 (PMC10308724; doi:10.1186/s40545-023-00582-9)
Supplement: Supplementary file 1 — Additional file 1: Table S1. Example search strategy- EMBASE. Table S2. Other clinical outcomes and their effects. [file 40545_2023_582_MOESM1_ESM.docx]

**Supplementary information**

**Table 1** Example search strategy- EMBASE

| 1. exp heart failure/ |
| --- |
| 2. heart failure*.mp. |
| 3. 1 or 2 |
| 4. exp medication compliance/ |
| 5. exp patient compliance/ |
| 6. "medication adherence".mp. |
| 7. (treatment adherence and compliance).mp. |
| 8. 4 or 5 or 6 or 7 |
| 9. exp telemedicine/ |
| 10. exp telehealth/ |
| 11. exp telemonitoring/ |
| 12. exp mobile application/ |
| 13. exp software/ |
| 14. exp mobile health application/ |
| 15. exp telephone/ |
| 16. exp text messaging/ |
| 17. exp electronics/ |
| 18. exp computer/ |
| 19. *mobile phone/ or *smartphone/ |
| 20. (tablet adj3 (device$ or computer$)).tw. |
| 21. mhealth*.mp. |
| 22. ehealth*.mp. |
| 23. SMS.mp. |
| 24. telecare*.mp. |
| 25. mobile health.mp. |
| 26. 9 or 10 or 11 or 12 or 13 or 14 or 15 or 16 or 17 or 18 or 19 or 20 or 21 or 22 or 23 or 24 or 25 |
| 27. 3 and 8 and 26 |
| 28. limit 27 to randomized controlled trial |

**Table 2:** Other clinical outcomes and their effects

| Study | Other clinical outcomes | Results | P-value(s) |
| --- | --- | --- | --- |
| Hale et al. [15] 2016, USA | Health (Self-reported  general health status)  Depression (PHQ-8)  NYHA stage  Quality of Life (MLHFQ)  (Higher score indicates worser quality of life)  Hospitalisations  ED visits | No significant difference in self-rate health.  No significant difference in depression.  Significantly poorer HF-related health.  Quality of life- mean MLHFQ score was 62.2 in the intervention versus 28.2 in control group.   Hospitalisation - number of participants who had one or more HF-related hospitalisations were 4 in intervention group versus 1 in control group and non-HF related hospitalisations were 1 in intervention group versus 4 in control group after 90 days. All-cause hospital admissions were significant, with 1 in intervention group versus 4 in control group.  ED visits - number of participants who had one or more HF-related ED visits were 1 in intervention group versus 3 in control group and non-HF related ED visits were 3 in intervention group versus 4 in control group after 90 days. All-cause ED visits were not significant, with 3 in the intervention group versus 6 in the control group. | Health = **0.820**  Depression = **0.460**  Quality of life = **0.002**  HF-related hospitalisations = **0.340**  non-HF-related hospitalisations = **0.340**  HF-related ED visits = **0.600**  non-HF-related ED visits = **0.990**  All-cause ED visits = **0.680**  All-cause hospitalisation days = **0.040** |
| Gallagher et al. [16] 2017, USA | 30-day all cause readmission  Attendance at follow-up  Acceptability of adherence telemonitoring | 30-day all cause readmission - number of participants hospitalised within 30 days were 6 for intervention group versus 4 in control group.  Follow-up attendance - number of participants who attended clinic appointment within 30 days were 19 for intervention group versus 16 in control group.  Acceptability of adherence telemonitoring - 90% of participants completed follow-up assessment. 88% rated the electronic pill bottle as somewhat or easy to us. 88% said they would use it again. | Rehospitalisation within 30 days = **0.720**  Rehospitalisation for HF within 30 days = **0.700**   Attendance at follow-up = **0.340**  Acceptability = **not available** |
| Goldstein et al. [17] 2014, USA | Mastery of the intervention (tested on ability to use the medication-related functions of the devices)  Device ratings- survey to assess participants acceptance of device (5-point scale and 14 items)  (Higher scores indicate higher rating for device) | Mastery of intervention - Study personnel- all participants demonstrated their knowledge of powering the device and its use.  Device ratings - mean score for device ratings were 48.7 for m-health intervention group versus 33.4 in telehealth intervention group. Participants preferred m-health intervention to telehealth intervention. | Mastery of the intervention = **not available**     M-health intervention over telehealth = **< 0.001** |
| Boyne et al. [18] 2014, Netherlands | Disease-specific knowledge (Dutch Heart Failure Knowledge Scale- 15 multiple-choice items)  (Higher scores indicate better knowledge)  Disease-specific self-care (European Heart Failure Self-Care Behaviour Scale)  (Lower scores indicate better results)   Disease -specific self-efficacy (Barnason Efficacy Expectation Scale)  Adherence to: Appointments Weighing Diet Fluid Activities Smoking Alcohol | Disease-specific knowledge – Mean score after 12 months follow-up was 13.5 for the intervention group versus 12.6 in the control group.  Self-care – mean score after 12 months follow-up was 17.4 for the intervention group versus 20.8 in the control group.  Self-efficacy – mean score after 12 months follow-up 54.9 for the intervention group versus 52.3. After correction for baseline score, significance disappeared.  Appointments – adherence to appointments at 12 months follow-up was 97.2% in the intervention group versus 97.3% in the control group.  Weighing – adherence to weighing at 12 months follow-up was 87.2% in the intervention group versus 72.8% in the control group.  Diet – adherence to diet at 12 months follow-up was 81.7% in the intervention group versus 80.9% in the control group.  Fluid – adherence to fluid restriction at 12 months follow-up was 84.8% in the intervention group versus 81% in the control group.  Activities – adherence to activities at 12 months follow-up was 65.8% in the intervention group versus 64.1% in the control group.  Alcohol – adherence to alcohol restriction at 12 months follow-up was 90.3% in the intervention group versus 92.5% in the control group. | Disease-specific knowledge = **< 0.001**  Disease-specific self-care = **< 0.001**   Disease-specific self-efficacy = **0.192**   Appointments = **0.981**  Weight = **0.000**  Diet = **0.724**  Fluid = **0.086**  Activities = **0.610**  Smoking = **0.918**  Alcohol = **0.311** |
|  |  |  |  |

**Table 2:** Other clinical outcomes and their effects (continued)

| Study | Other clinical outcomes | Results | P-value(s) |
| --- | --- | --- | --- |
| Wu et al. [19] 2012, USA | Cardiac event-free survival (Including ED visit, hospitalization, death)  Quality of life (Minnesota Living with Heart Failure Questionnaire) | Significant difference on event-free survival among 3 groups (PLUS and LITE did not differ so data was combined).  Event-free survival was significantly longer for the patients in both intervention groups than the control.  Mean score for quality of life at 90 days follow-up for intervention group (PLUS) was 32.9 for intervention group versus 40.1 in control group. | Event free survival (all 3 groups) = **0.034**  Event-free survival = **0.010**  Quality of life = **not available** |
| Felker et al. [20] 2022, USA | Change in mean daily step count from baseline through 3 months  Quality of life (Kansas City Cardiomyopathy Questionnaire)  (Higher score indicates better quality of life)  Physiological measure of disease status (NT-proBNP, HbA1C) | Change in mean daily step count – changes in step count at 3 months follow-up were 151 in the intervention group versus -162 in the usual care group. Significant difference between groups (difference 313 with 95% CI [8, 619]).   Quality of life – mean score at 3 months follow-up was 6.6 in the intervention group versus 1.1 in the control group. Significant difference between groups (difference 1.1 with 95% CI [1.4, 9.6]).   Change in mean NT-proBNP – change in mean NT-proBNP at 3 months follow-up was -41 in in the intervention group versus 24 in the control group. Treatment difference was -65 with 95% CI [-164, 34].   Change in HbA1c – change in HbA1c at 3 months follow-up as a percentage was 0.13% in the intervention group versus -0.02 in the control group. Treatment difference was 0.15 with a 95% CI [-0.24, 0.55].  13 metabolites showed significant results for differential change from baseline- majority of which were medium- and long-chain acylcarnitine’s. | Mean daily step count = **0.044**  Quality of life = **0.009**  NT-proBNP = **0.200**  HbA1C = **0.440**  Metabolites = **not available** |
| Yanicelli et al. [21] 2020, Argentina | Changes in self-care (European Heart Failure Self-Care Behaviour Scale)  Rehospitalisation (from electronic medical record) | Mean self-care score at 3 months follow-up was 80.03 for intervention group versus 69.43 in control group.  Number of rehospitalisation were 0 for intervention group and 2 in control group after 3 months follow-up. | Self-care = **0.004**  Re-hospitalisations = **0.500** |
| Young et al. [22] 2016, USA | Other self-management adherence  Physical activity (ActiGraph)  Clinical biomarkers  All-cause readmissions  Emergency Department visits | Mean number of days for weighing self per week was 4.8 in the intervention group versus 1.9 (control) at 3 months and 4.6 (intervention) versus 1.5 (control) at 6 months. Estimated marginal mean was 4.7 (intervention) versus 1.7 (control). 95% CI: 2.98 [2.10, 3.86].  Mean number of days for following low-sodium diet per week was 5.6 in the intervention group versus 3.1 (control) at 3 months and 5.1 (intervention) versus 2.3 (control) at 6 months. Estimated marginal mean was 5.3 (intervention) versus 2.7 (control). 95% CI: 2.62 [1.74, 3.50].  Mean number of days for exercising per week was 5.4 in the intervention group versus 3.4 (control) at 3 months and 4.5 (intervention) versus 3.1 (control) at 6 months. Estimated marginal mean was 4.9 (intervention) versus 3.3 (control). 95% CI: 1.66 [0.79, 2.53].  Mean average daily activity counts were 285,707 in the intervention group versus 251,265 (control) at 3 months and 306,648 (intervention) versus 250,913 (control) at 6 months. Estimated marginal mean was 279,160 (intervention) versus 270,924 (control). 95% CI 8236 [-50,155, 66,628].  Mean average daily activity kcals/kg/day were 2.2 in the intervention group versus 1.8 (control) at 3 months and 2.3 (intervention) versus 1.7 (control) at 6 months. Estimated marginal mean was 2.08 (intervention) versus 2.01 (control). 95% CI 0.07 [-0.42, 0.56]. | Weighing = **<0.005** Low sodium = **<0.005** Exercise = **<0.005** BNP = **0.282** Average daily sodium intake = **0.234** All-cause readmissions = **0.088** Average daily activity counts = **0.780** Average activity kcals = **0.773** Average daily min doing moderate activity = **0.897** Self-management knowledge = **0.337** Self-efficacy for HF self-management = **0.034** Patient activation = **0.069** Self-management = **< 0.005** |

**Table 2:** Other clinical outcomes and their effects (continued)

| Study | Other clinical outcomes | Results | P-value(s) |
| --- | --- | --- | --- |
|  |  | Mean average daily minutes doing moderate intensity or more activity was 5.8 in the intervention group versus 3.3 (control) at 3 months and 5.9 (intervention) versus 3.7 (control) at 6 months. Estimated marginal mean was 4.81 (intervention) versus 4.65 (control). 95% CI 0.17 [-2.34, 2.71].  B-type natriuretic peptide was 1.7 in the intervention group versus 1.8 (control) at 3 months and 1.7 (intervention) versus 1.8 (control) at 6 months. Estimated marginal mean was 1.7 (intervention) versus 1.8 (control). 95% CI -0.09 [-0.26, 0.08].  Average daily sodium intake was 3607 mg in the intervention group versus 3876.9 mg (control) at 3 months and 3748.7 mg (intervention) versus 3926.6 mg (control) at 6 months. Estimated marginal mean was 3647.9 mg (intervention) versus 3919.9 mg (control). 95% CI -272.03 [-722.70, 178.65].  Number of participants readmitted to hospital was 10 for intervention group versus 3 in the control group.  Number of participants admitted for ED visits at 90 days were 9 for intervention group versus 11 in control group. |  |
| Ross et al. [23] 2004, USA | Self-efficacy (Kansas City Cardiomyopathy Questionnaire)  Patient satisfaction (Art of Medicine Questionnaire)  General adherence to medical regimens (General Adherence Scale from the Medical Outcomes Study (MOS)) | No statistically significant improvements were demonstrated in health status when adjusted for multiple comparisons.  Significant results were found in patient satisfaction, but findings did not reach statistical significance when adjusted for multiple comparisons.  Mean score for general adherence was 85 in the intervention group versus 78 in control group. 95% CI +6.4 [1.8, 10.]. | Self-efficacy = **0.080, 0.060, 0.960, 0.630, 0.310, 0.380, 0.260 (each question)**  Patient satisfaction = **0.130, 0.130, 0.150, 0.260, 0.800, 0.300 (each question)**  General adherence = **0.020** |
